# Supplementary figures and images for: Is it worth the extra mile? Comparing environmental DNA and RNA metabarcoding for vertebrate and invertebrate biodiversity surveys in a lowland stream
Source: PeerJ. 2024 Oct 24;12:e18016. doi: 10.7717/peerj.18016 (PMC11512801; doi:10.7717/peerj.18016)

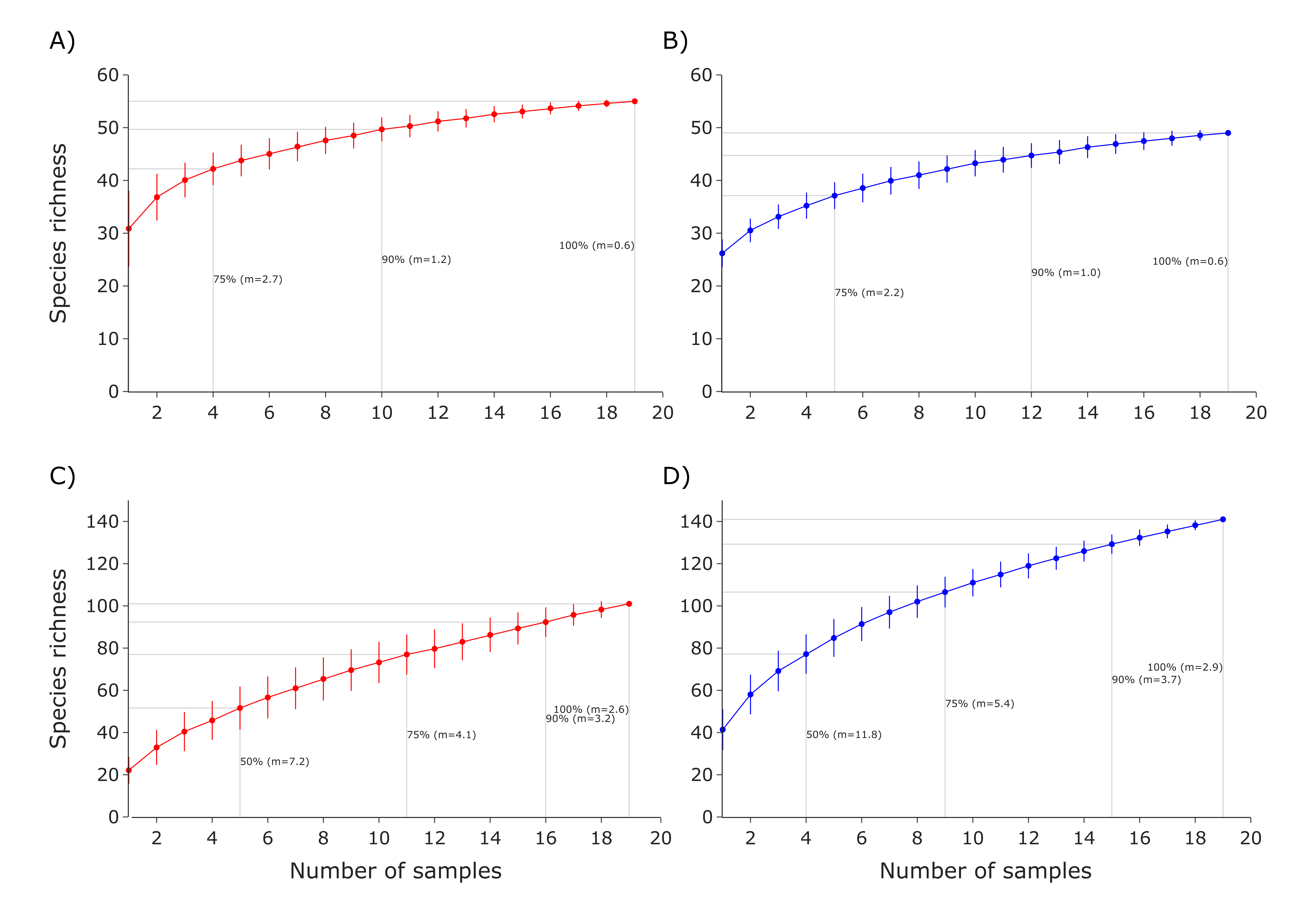

Supplement: Supplemental Information 1 — Up to four intersections (50%, 75%, 90%, and 100%) with the respective slope (m) are highlighted for each rarefaction curve. [file peerj-12-18016-s001.png]

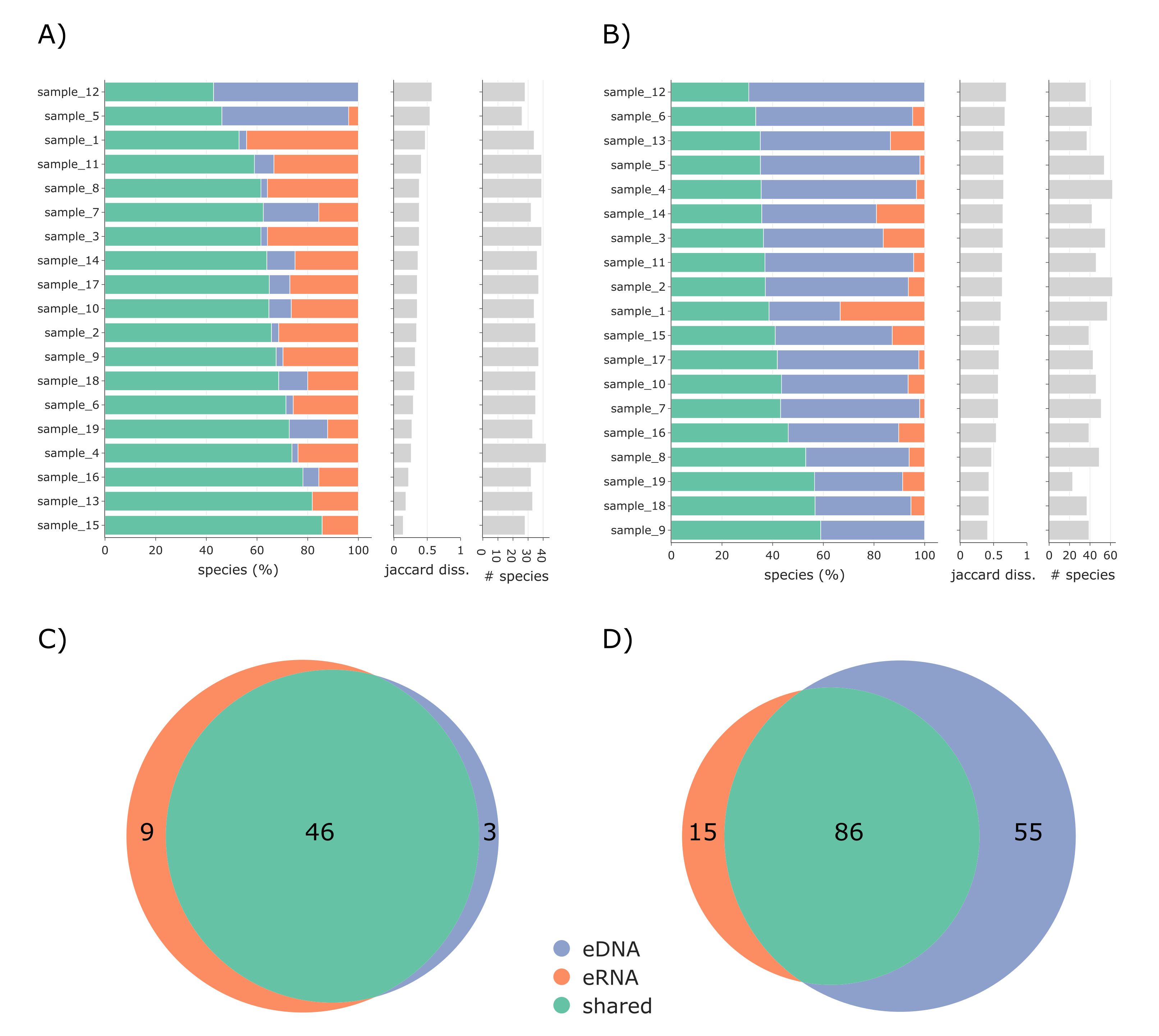

Supplement: Supplemental Information 2 — Pairwise comparison of eDNA-based (blue) and eRNA-based (red) exclusive and shared (green) species for vertebrates (A) and invertebrates (B). Samples are sorted by Jaccard dissimilarity. The overall numbers of shared and exclusive species for the vertebrate (C) and invertebrate (D) datasets are displayed as Venn diagrams. [file peerj-12-18016-s002.png]

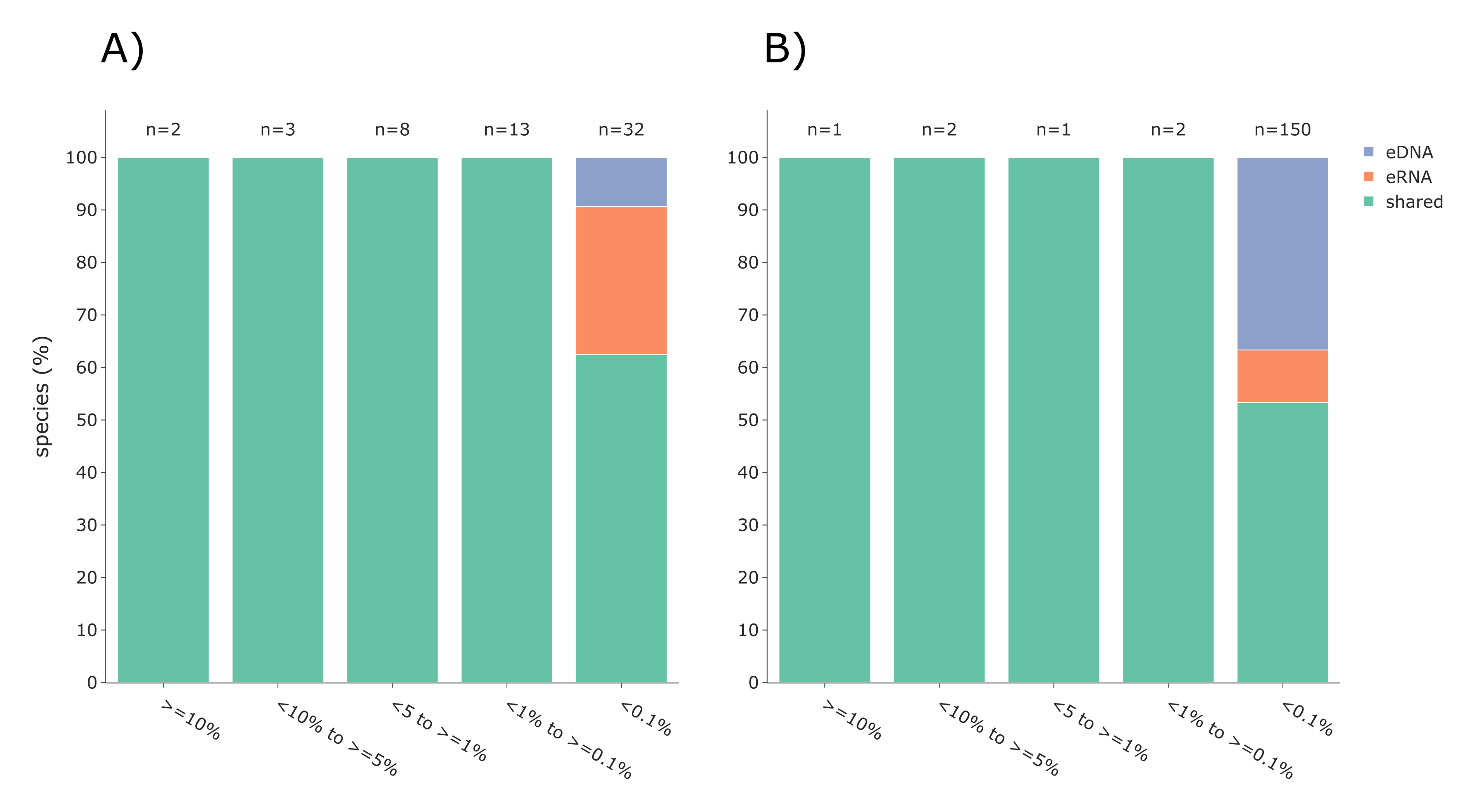

Supplement: Supplemental Information 3 — Species were sorted according to their relative read abundance into five categories. The number of species per category is given above the respective bar. [file peerj-12-18016-s003.png]

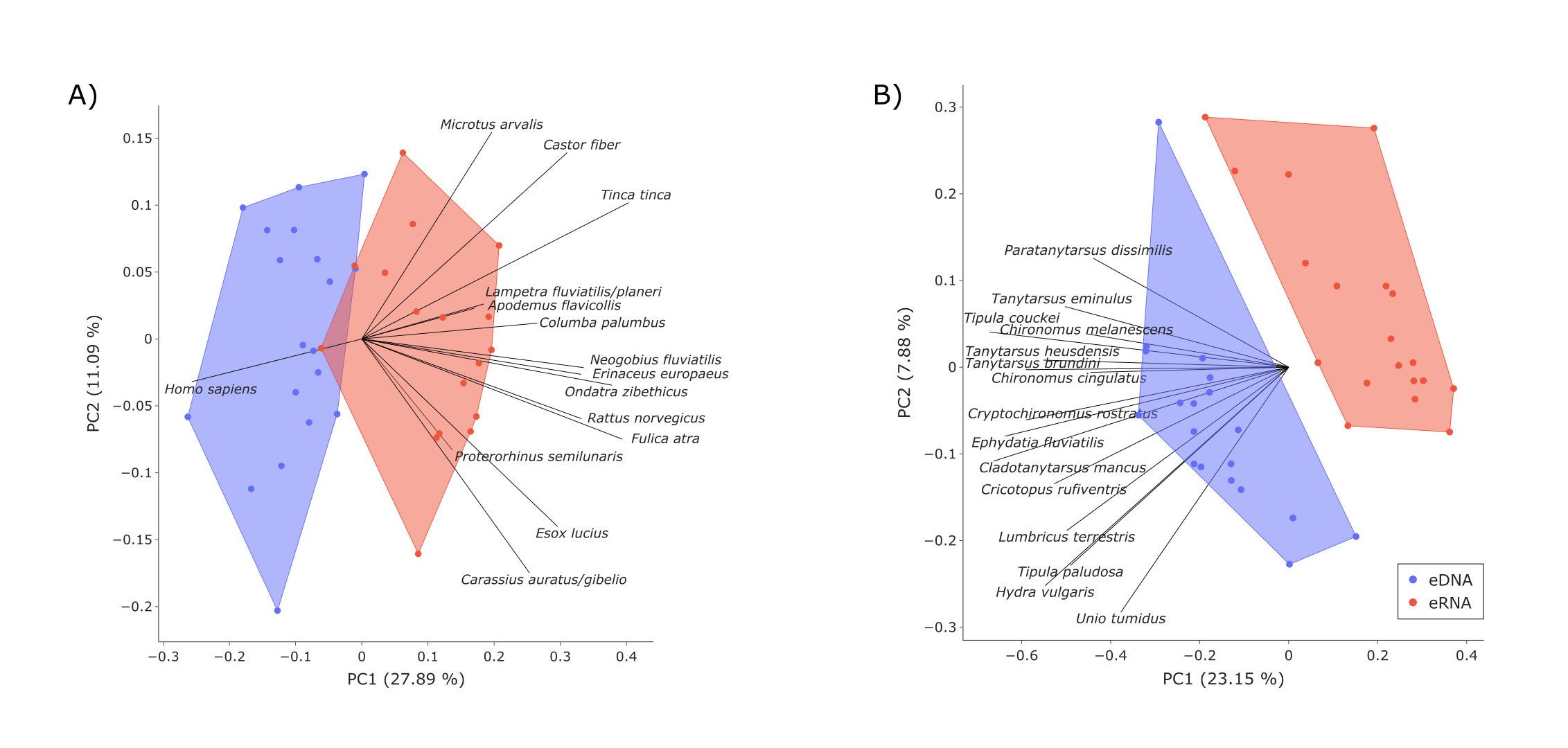

Supplement: Supplemental Information 4 — The principal coordinate analysis (PCoA) revealed group trends for vertebrates (Anosim R = 0.5, p = 0.001; samples ‘eRNA_5′and ‘eRNA_12′were identified as outliers and excluded from the PCoA). PCoA of the invertebrate dataset revealed distinct groups of eDNA and eRNA samples (Anosim R = 0.55, p = 0.001). The four most representative species per quadrant are shown (logistic regression analysis). [file peerj-12-18016-s004.png]

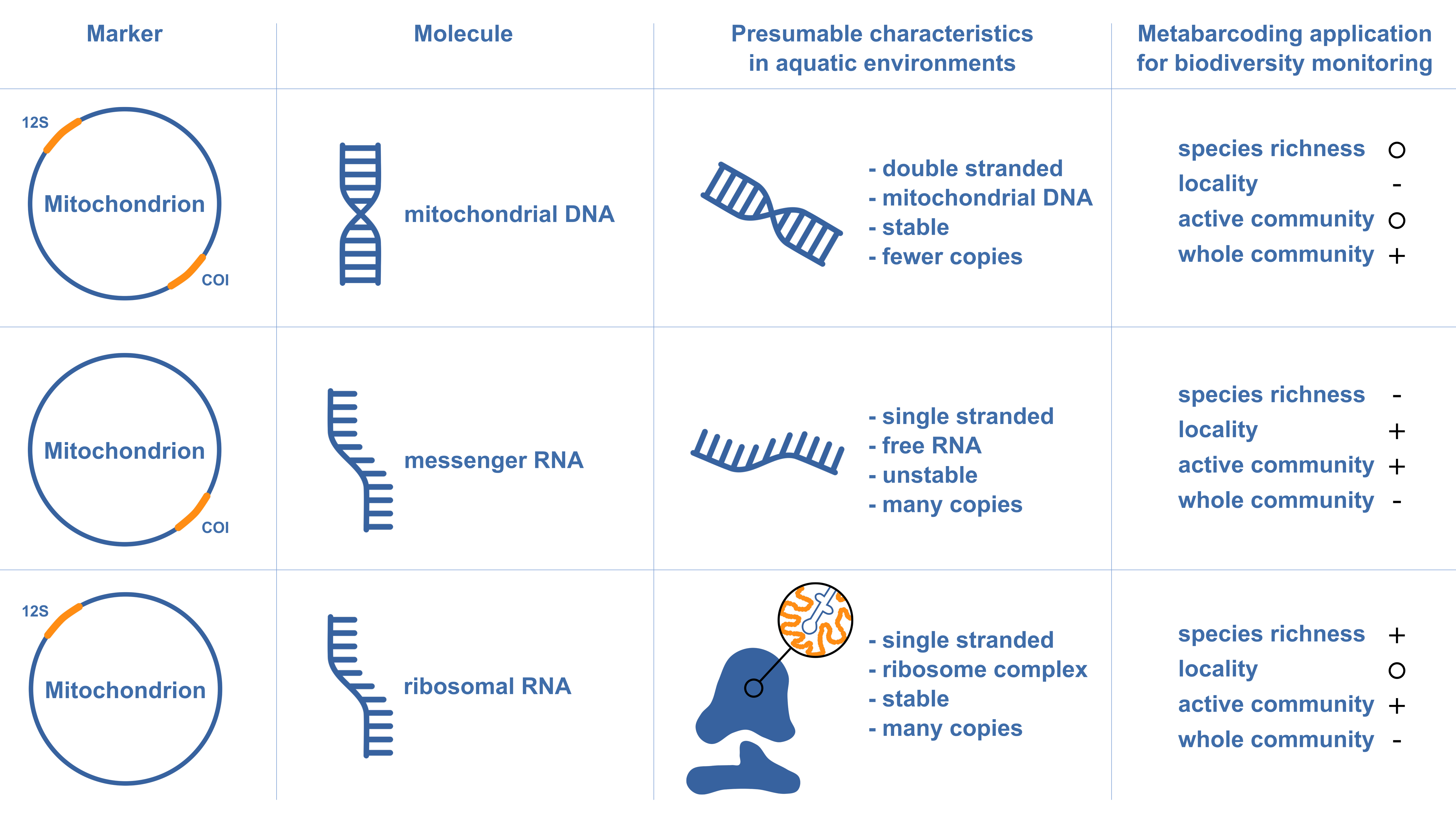

Supplement: Supplemental Information 5 — Different RNA molecules behave differently in the environment due to their biology, impacting their use in biodiversity monitoring. We evaluated and compared options for biodiversity monitoring based on marker and molecule with high (+), neutral (circle), and low (+). [file peerj-12-18016-s005.png]
